# A noninferiority randomized open-label pilot study of 3- versus 7-day influenza postexposure prophylaxis with oseltamivir in hospitalized children

**DOI:** 10.1038/s41598-024-65244-5

**Published:** 2024-06-20

**Authors:** August Wrotek, Teresa Jackowska

**Affiliations:** 1 Department of Pediatrics, The Centre of Postgraduate Medical Education, Warsaw, Poland; 2 Department of Pediatrics, Bielanski Hospital, Warsaw, Poland

**Keywords:** Oseltamivir, Antiviral agents, Infection control, Chemoprevention, Influenza, Virology, Influenza virus

## Abstract

Short influenza postexposure prophylaxis (PEP) showed high efficacy in adults, but studies in children are lacking. This randomized open-label pilot trial aimed to verify noninferiority of a 3- versus 7-day prophylaxis with oral oseltamivir in hospitalized children. Influenza contacts were randomized to the 3- or 7-day group and efficacy, relative risk of adverse events (AEs), and the cumulative costs of drugs and AEs management were compared. The intention-to-treat (ITT) analysis included 59 children (n = 28 and n = 31 in the 3- and 7-day group, respectively). The efficacy was 100% (95% CI 87.7–100%) versus 93.6% (95% CI 78.6–99.2%) in the 3- and 7-day group; the differences were statistically insignificant. A per-protocol (PP) analysis including 56 patients (n = 27 and n = 29, respectively) showed 100% (95% CI 87.2–100%) and 93.1% (95% CI 77.2–99.2%) efficacy, respectively, without statistical significance. Differences were within the predefined noninferiority margin with an efficacy difference Δ = 6.45 percentage points (p.p.) with 1-sided 95% CI (− 2.8, − 1.31, *p* = 0.86; ITT) and Δ = 6.9 p.p. (1-sided 95% CI − 2.83, − 1.27, *p* = 0.85; PP). Adverse events did not differ significantly, while the cumulative costs of the prophylaxis and AEs management were higher in the 7-day group (median 10.5 euro vs. 4.5 euro, *p* < 0.01). This pilot study showed the noninferiority of the 3-day versus 7-day PEP, which was associated with lower costs.

Trial registration number: NCT04297462, 5th March 2020, restrospectively registered.

## Introduction

Influenza is one of the major public health concerns, and the pediatric population is of particular interest due to the high morbidity, hospitalisation risk and high number of complications^1–4^. Influenza attack rate varies between 8.7%^5^ and 15.2%^1^ in children, with estimates for symptomatic and asymptomatic influenza combined reaching 22.5%^6^. The disease course may vary from mild to fatal, with the highest mortality observed in the youngest group of patients^7,8^. Except for age, a variety of underlying health conditions are recognised risk factors for severe influenza; while early use of antivirals in the case of hospitalization has been associated with shorter hospitalization, decreased risk of readmission, intensive care or death in children, there still exist many barriers impeding treatment implementation even in the vulnerable population^9–11^. Although vaccines have been available for many years, and a vaccination is the method of choice for influenza prevention, in the case of the need for an urgent outbreak control in a hospital, low vaccine coverage and uncertainty on vaccine effectiveness might make this measure suboptimal^12,13^.

The postexposure prophylaxis (PEP) may be indicated by individual patient’s characteristics (e.g. patients in high-risk group of a severe disease course) or epidemiological reasons^14^. In hospitalized children antiviral prophylaxis may be driven by both individual indications or hospital outbreak control measures, which is recommended if two or more nosocomial laboratory confirmed cases are diagnosed at the same ward within 72 h^15,16^. Two neuraminidase inhibitors (NAIs) may be used in the chemoprophylaxis in children: oral oseltamivir and inhaled zanamivir, which have been shown to be effective both in the treatment as well as in the prophylaxis of influenza^17–20^. NAIs show a 70–90% efficacy in influenza prevention, and the timing is crucial – NAIs are not recommended if the time since the first exposition to the influenza virus exceeds 48 h^15^. Oseltamivir use is recommended by the Centers for Disease Control and Prevention (CDC) and the American Academy of Pediatrics (AAP) in children aged 3 months of age or more for prophylaxis and in critical situations for younger infants; zanamivir, on the other hand, is licensed in older children (5 years of age and above), which significantly restricts its use in clinical practice^15^.

The safety profile of oseltamivir seems to be satisfactory; its use correlated in children with a higher risk of vomiting, without an increased risk of other adverse events (AE), including those observed in adult patients (nausea, renal events, and psychiatric effects)^18^. Irrespectively of the good safety profile, the shortest possible (and long enough to guarantee efficacy) exposition to the drug is the preferred approach, and the recommended duration of PEP varies between 7 to 10 days after the last known exposure^15,19^. The study by Ishiguro et al. (2016) analyzed the efficacy of a 3-day PEP course, showing high overall efficacy, comparable to that of longer (7–10-day) regimens (93%, with a 95% confidence interval: 53%–99%; p = 0.023)^21^. The study included several pediatric patients (the exact number has not been specified), and inspired us to perform such an analysis on the pediatric population^21^.

In this randomized open-label trial, we aimed to compare the efficacy, safety, and costs of a 3- versus 7-day influenza prophylaxis with oral oseltamivir in hospitalized children.

## Material and methods

### Study design and setting

This prospective randomized open-label study is a pilot noninferiority investigation comparing the efficacy of shorter (3 days) and longer (7 days) influenza postexposure chemoprophylaxis with oseltamivir in a hospital setting. The trial was conducted at the Pediatric Ward of the Bielanski Hospital, Warsaw, Poland. Influenza contacts, i.e. children hospitalized due to other than influenza causes who had been contacted with influenza index case(s) during their hospitalization were eligible for the study. The trial was approved by the local Ethics Committee at The Centre of Postgraduate Medical Education (the permission number 77/PB/2016 issued on 16th November 2016). The trial was registered in the public clinical trial register at clinicaltrials.gov with the identifier NCT04297462. The study was registered after the patient enrolment had commenced, which was in accordance with the local legal regulations that permit the trial registration to be voluntary during the whole period of the patient’s enrolment if an appropriate Ethics Committee approval is granted.

### Definitions

#### Influenza index case

Only patients with laboratory confirmed influenza were considered to be the index cases. Patients were tested towards influenza in the case of a clinical suspicion, i.e. the signs or symptoms of an influenza-like illness (ILI) or a presentation other than respiratory that could be attributable to an influenza infection (e.g., neurological manifestations, including febrile seizures)^22^. The presence of the virus was confimed in the patient’s nasopharyngeal swab with the use of the rapid influenza diagnostic test (RIDT) or the Reverse Transcription-Polymerase Chain Reaction (RT-PCR); the latter one was considered conclusive in the case of any discrepancy. The following RIDTs were used: Nadal Influenza A + B cassette (nal von minden GmbH, Moers, Germany), BinaxNOW Influenza A&B Card (Alere Scarborough Inc., Scarborough, Maine, USA), BD DIRECTIGEN EZ FLU A + B (Becton, Dickinson and Company, Sparks, Maryland, USA), while RT-PCR was performed with a cartridge based nucleic acid amplification test Cepheid GeneXpert (Sunnyvale, California, USA). Each index case was given antiviral treatment at the time of the diagnosis in accordance with the CDC guidelines^15^.

#### Influenza Contact

Each patient hospitalized in the same room with an influenza index case was identified as influenza contact (the minimal duration of the contact was 8 h). Due to the different room capacities, cubatures, and varying number of persons per one room (between two to four patients and one or no parent/tutor per patient), alongside with the different patterns of the children’s behaviour, no distinction between direct (e.g. playing or speaking with the index case) and indirect contacts was made. This approach is in line with the study by Shinjoh et al. who included both the patients with direct contact and with close contact (admitted to the same room ass the index case)^23^. Due to the local legal regulations, no PEP could have been ordered directly by the pediatric ward’s staff to the parents or tutors present in the same room with the children, however, in each case of an influenza contact, a general practitioner’s visit was recommended, and an appropriate letter to the general practitioner was issued. In case any signs or symptoms should emerge, any symptomatic person (except for the patients) was asked to leave the hospital as soon as they noticed the first symptoms, and the child was then looked after by another parent/tutor or by the pediatric ward staff.

#### Patients’ allocation

A written informed consent of a parent/legal tutor and a patient (aged 16 years or more, according to the Polish law regulations) was obtained before the randomization. Once the consent was given, patients were randomized according to the previously generated table of randomization into the 3- or 7-day PEP group, the allocation ratio was 1:1 with block size ≤ 3. The table was generated using a table generator and sequentially numbered randomization results were opened at patient enrolment. The random allocation sequence was generated by A.W., while patient enrolment and allocation to the intervention group was performed by a local study team.Patients have their inherent right to discontinue the study participation at any moment. Enrolment was stopped when the calculated minimum number of patients was reached.

#### Postexposure prophylaxis and follow-up

All the contacts were given oral oseltamivir in accordance with the local Polish recommendations for hospital prophylaxis, i.e. a single day dose of 30mg, 45mg, 60mg or 75mg in children aged 1 year old or more (weight range: up to 15kg; > 15–23kg; > 23- 40 kg, and > 40kg, respectively), or 3mg/kg/dose once a day in children aged 3–12 months old, 2.5mg/kg/dose in children aged 1–2 months, and 2mg/kg/dose in neonates) (10). Oseltamivir was given during hospitalization, and if the PEP duration exceeded the period of hospitalization, a continuation of the prophylaxis (up to 3 or 7 days, depending on the randomization result) was recommended. A follow-up lasted 7 days after the PEP had stopped: patients hospitalized during that period of time were examined daily, and if any signs or symptoms of a possible influenza were observed, an RT-PCR in a sample from a nasopharyngeal swab was performed immediately. Similarly, each discharged patient was asked to report immediately any signs or symptoms of influenza-like illness if they emerged during the PEP and within 7 days after the PEP had finished., and an RT-PCR was performed if patients presented with at least one of the symptoms: fever, cough, headache , muscle pain, general feeling of ill-health or sore throat. During the week following the end of the PEP, an additional passive inquiry was conducted regarding influenza signs and symptoms.

#### Adverse events data collection

Parents/tutors/patients were asked to report any of the following adverse events (AE): nausea, vomiting, stomachache, headache, behavioral changes, including excitation and apathy, sleep disorders, skin hypersensitivity (including rash) in a prospective manner. After the discharge from the hospital, parents/tutors were asked about the presence of the AEs mentioned above up to 7 days after finishing the PEP.

#### Outcomes

Primary outcome measures included the PEP efficacy, safety (risk of adverse events in both groups), and the cumulative costs of drugs in each arm of the study, as well as the cost of the management of the adverse events related to the drug administration. In order to avoid the influence of the weight on the costs, we calculated the mean cost of the oseltamivir prophylactic dose, adding the cost of the appropriate 30 mg, 45 mg, 60 mg, and 75 mg doses (in the case of a dose lower than 30 mg, the cost of the 30 mg dose was used) and dividing it by the number of patients; then the mean cost was multiplied by the number of doses (3 or 7). The cost of the adverse events was estimated per AE episode: each AE episode was assumed to bring the risk of additional medical attendance, the official cost of an ambulatory visit for uninsured patients was used^24^. The costs were calculated and converted into EUR as of 31.12.2020^25^.

Secondary endpoints included the differences between 2 PEP regimens related to influenza complications: need for hospitalization in the case of a failed influenza chemoprophylaxis (i.e., if influenza was present within 7 days after the PEP completion), the duration of the influenza signs and symptoms, including fever after a failed chemoprophylaxis, the presence of complications in the case of a failed influenza chemoprophylaxis (pneumonia, bronchitis, otitis media, the need for antibiotic treatment, neurological sequelae, ICU transfer, death). The time-frame for secondary endpoints analysis was 28 days.

### Statistical analysis

Sample size calculation was based upon the assumption of a 99% efficacy according to the results from the previous study by Ishiguro^21^. A minimal sample size for the noninferiority of the 3 versus the 7-day PEP with a 90% power, α = 0.05 and a noninferiority border of 8 percentage points reached 27 patients per study arm. Two types of analysis were performed: intention-to-treat (ITT) and per-protocol (PP); for the ITT analysis, all patients originally enrolled in the trial were included; for the PP analysis, only patients who strictly adhered to the protocol were eligible.

The data distribution was assessed with the Kolmogorov–Smirnov test. Categorical data were presented as numbers (n) and frequency (%) and chi-square test was used for comparisons,

while continuous data were presented, depending on the distribution, as a mean and a standard deviation (SD) or a median and an interquartile range (IQR) for normally or not-normally distributed variables, respectively. A corresponding parametric or non-parametric test was used for direct comparisons (the Student’s t-test or the Mann–Whitney U test). The z-test was used to assess the efficacy with 95% confidence intervals observed in the whole prophylactic group.. A difference between efficacy of the 3-day and 7-day PEP regimen (Δ) was estimated with the use of 1-sided 95% CI and the noninferiority of the 3-day regimen was recognized if the lower limit of Δ 95% CI did not exceed − 8 percentage points (p.p.). Since the advantages of the PEP seem to be unquestionable, we did not create a no-prophylaxis group, and used the results from the study by Ishiguro as a reference for no-prophylaxis group (there, 13.3% breakthrough influenza cases were reported without PEP)^21^. Relative risks of adverse events with 95% confidence intervals (95% CI) were calculated. The ITT and PP populations were analyzed separately. The statistical significance was recognized for the p-value under 0.05. The statistical analysis was performed with the Statistica 13.1 software (TIBCO, Santa Clara, CA, USA, https://www.statsoft.pl/statistica_13/).

### Ethics approval

This study was performed in line with the principles of the Declaration of Helsinki. Approval was granted by the Ethics Committee of the Centre of Postgraduate Medical Education in Warsaw (the permission number 77/PB/2016 issued on 16th November 2016).

### Consent to participate

Informed consent was obtained from legal guardians of all individual participants included in the study.

## Results

### Study group

During the study period between December 2016 and February 2020, 60 patients were included and randomly allocated into the 3- or 7-day prophylaxis group (n = 29 or n = 31 patients, respectively).

**Figure 1:**
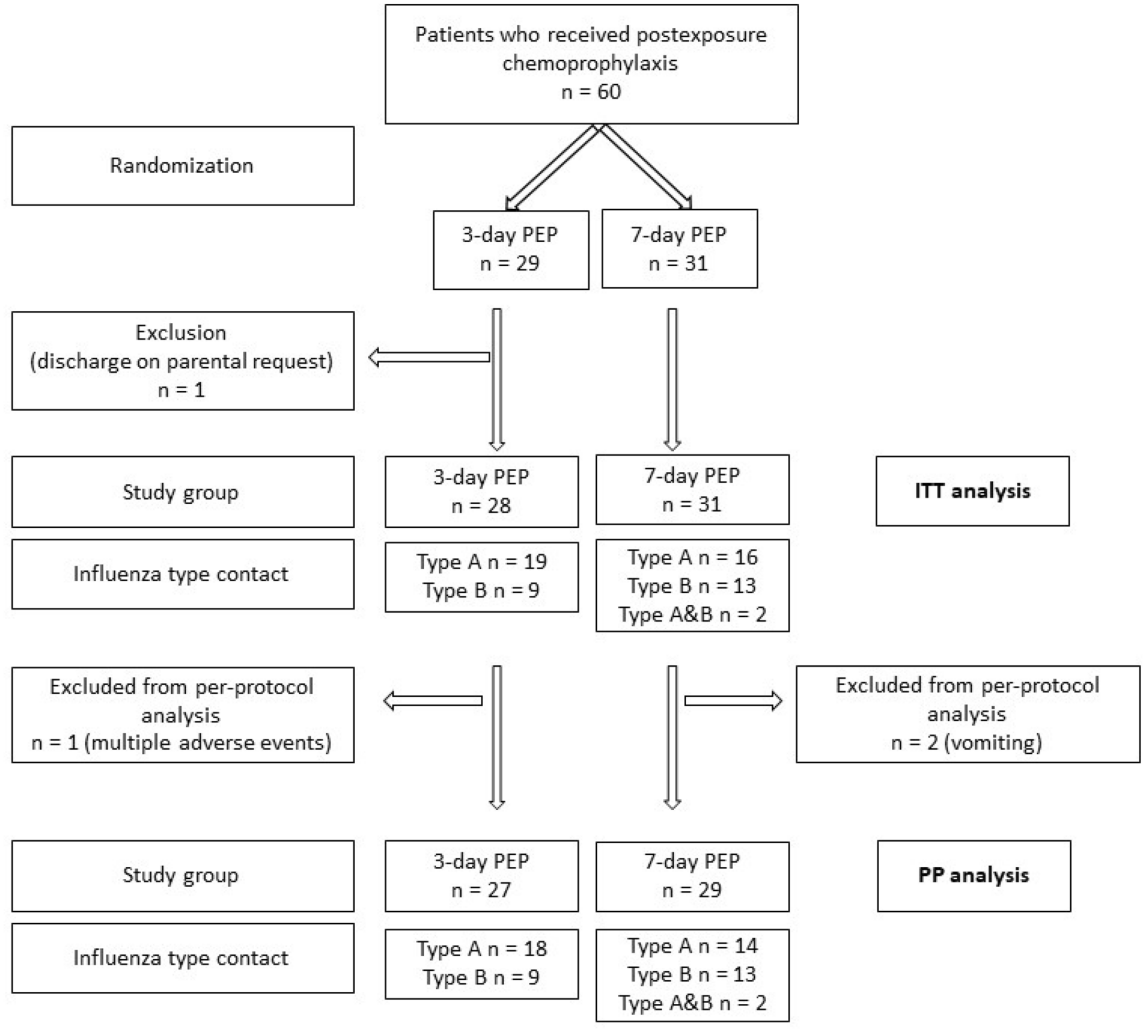
A flowchart of the patients in the study (abbreviations: n- number of patients, mo- months old, PEP- postexposure prophylaxis, ITT- intention-to-treat, PP- per-protocol).

The final ITT group consisted of 59 patients (28 and 31, respectively) aged from 10 days to 16 years and 1 month (median 23 months), one patient was excluded due to the discharge on parental request and lack of follow-up data (Fig. 1).

There were 3 cases of a withdrawal, one took place in the 3-day group (1-month old infant on the 2nd day) and two cases in the 7-day group (a 21-month old patient on the 4th day of the PEP, and a 56-month old patient on the 3rd day of the regimen). They were related to a poor drug tolerance: multiple adverse events (vomiting, sleep/behavioural changes, skin disorder) reported by the parents in an infant from the 3-day group and vomiting in patients from the 7-day group; the difference in withdrawal rate remained statistically insignificant (*p* = 0.62). The final PP group included 56 patients (27 and 29 in the 3- and 7-day prophylaxis arm, respectively).

The study groups differed in terms of the median age both in the ITT group (14.5 in the 3-day group vs. 26.5 months in the 7-day group, *p* = 0.02) and the PP group (14.6 vs. 26.5, respectively, *p* = 0.036); as consequence, there were differences in body weight, but not in oseltamivir dose per body weight (Table 1).

**Table 1:** Baseline characteristics of the study groups (3-day vs. 7-day group). Abbreviations: ITT- intention-to-treat, PP- per-protocol, PEP- postexposure prophylaxis, n- number of patients, IQR- interquartile range, N/A—not applicable.

Characteristic	ITT (n = 59)			PP (n = 56)		
	3-day PEP (n = 28)	7-day PEP (n = 31)	p	3-day PEP (n = 27)	7-day PEP (n = 29)	p
Gender: male/female [N] (% of male)	17/11 (60.7)	20/11 (64.5)	0.76	17/10 (62.9)	18/11 (62.1)	0.95
Age [months] median and (IQR)	14.5 (2.8–34.2)	26.5 (17.2–58.5)	0.02	14.6 (2.9- 35.1)	26.5 (17.2–58.5)	0.04
Body weight [kg] median and (IQR)	8.6 (6.1–15.4)	15.1 (11.2–18.6)	0.01	8.8 (6.4- 15.5)	15.2 (10.3- 19.9)	0.02
Oseltamivir dose per body weight [mg/kg] median and (IQR)	2.86 (2.6–3)	2.56 (2.3–2.9)	0.13	2.87 (2.6- 3)	2.56 (2.3- 2.9)	0.13
Influenza vaccination-current season	0/0	0/0	N/A	0/0	0/0	N/A
Influenza vaccination-pregnancy	0/0	0/0	N/A	0/0	0/0	N/A
Length of stay [days] median and (IQR)	5.5 (4–9)	6 (5–8)	0.68	6 (4–9)	6 (5–8)	0.70
Withdrawal [N] (%)	1 (3.6%)	2 (6.5%)	0.62	N/A	N/A	N/A

### Epidemiological data

The patients in the ITT group had contact with influenza A in 35 cases (19 cases in the 3-day and 16 in the 7-day group), with influenza B in 22 cases (9 and 13 cases in respective groups), and with both A and B types in 2 cases from the 7-day group. The PP group patients had contact with influenza A in 32 cases (18 and 14 cases, respectively), with influenza B in 22 cases, and with influenza A and B types in 2 cases. None of the patients had previously been vaccinated against influenza, neither had any of the mothers been immunized against flu during pregnancy.

### Efficacy of postexposure prophylaxis

Two patients developed respiratory tract infections due to laboratory-confirmed influenza type A (n = 1) and type B (n = 1). No failures of the PEP were observed in the 3-day group, while both failures took place in the 7-day group (influenza type A on 4th day of prophylaxis, and influenza type B on 6th day of prophylaxis).

The general PEP efficacy in the ITT analysis reached 96.6% (95% CI 88.3% to 99.6%) (57/59), while in the PP analysis it was 96.4% (95% CI 87.6% to 99.6%) (54/56) (Table 2). Since low influenza incidence was observed in both the 3-day and 7-day groups, a comparison with the assumed efficacy of no PEP (86.7% according to the literature) showed a statistically significant advantage of PEP over no prophylaxis with a z-score of 2.24 (p = 0.025) in the ITT analysis and 2.2 (*p* = 0.033) in the PP analysis.

**Table 2:** A summary of efficacy and safety of influenza postexposure prophylaxis and a comparison of the study groups (3-day vs. 7-day group) in the intention-to-treat and per-protocol analysis. The relative risk represents the risk between the 3-day and the 7day PEP group. Abbreviations: ITT- intention-to-treat, PP- per-protocol PEP- postexposure prophylaxis, 95% CI- 95-percent confidence interval, n- number of patients, IQR- interquartile range.

	ITT (n = 59)			PP (n = 56)		
	3-day PEP (n = 28)	7-day PEP (n = 31)	p	3-day PEP (n = 27)	7-day PEP (n = 56)	p
General PEP efficacy [%] (95% CI)	96.6% (88.3% to 99.6%)			96.4% (87.6% to 99.6%)		
PEP efficacy [%] (95% CI)	100% (87.7–100%)	93.55% (78.6–99.2%)	0.86	100% (87.2– 100%)	93.1% (77.2–99.2%)	0.85
Adverse events (total) [n] (%)	21 (35.6)			18 (32.1)		
Adverse events [n](%)	8 (28.6)	13 (41.9)		7 (26)	11 (38)	
Relative risk (95% CI)	0.68 (0.33–1.4)		0.29	0.68 (0.31–1.5)		0.35
Vomiting [n] (%)	3 (10.7)	9 (29)		2 (7)	7 (24)	
Relative risk (95% CI)	0.37 (0.11–1.23)		0.1	0.31 (0.07–1.35)		0.12
Behavioural changes [n](%)	4 (14.3)	2 (6.5)		3 (11)	2 (7)	
Relative risk (95% CI)	2.2 (0.44–11.2)		0.34	1.61 (0.29–8.91)		0.59
Stomachache [n](%)	1 (3.6)	3 (9.7)		1 (4)	3 (10)	
Relative risk (95% CI)	0.37 (0.04–3.35)		0.38	0.36 (0.04–3.24)		0.36
Skin disorders [n](%)	3 (10.7)	1 (3.2)		2 (7)	1 (3)	
Relative risk (95% CI)	3.32 (0.37–30.12)		0.29	2.14 (0.21–22.36)		0.52
Sleep disorders [n](%)	2 (7.1)	0 (0)		1 (4)	0 (0)	
Relative risk (95% CI)	5.52 (0.28–110.21)		0.26	3.21 (0.14–75.68)		0.47
Total multiple adverse events [n](%)	5 (8.5)			4 (7.1)		
Multiple adverse events [n](%)	3 (10.7)	2 (6.5)		2 (7)	2 (7)	
Relative risk (95% CI)	1.67 (0.3–9.23)		0.56	1.07 (0.16–7.1)		0.94

The efficacy ob both the 3-day and 7-day PEP was high in the ITT analysis, reaching 100% (95% CI 87.7–100%) and 93.6% (95% CI 78.6–99.2%), respectively, while in the PP analysis the efficacy was similarly high with 100% (95% CI 87.2–100%) and 93.1% (95% CI 77.2–99.2%) in the 3-day and 7-day PEP groups, respectively. The difference between the 2 PEP regimens, however, remained statistically insignificant in both ITT and PP analyses.

The ITT group difference between the efficacy of the 3-day and 7-day regimens (100% and 93.55%, respectively) equalled 6.45 p.p. with 1-sided 95% CI reaching (− 2.8, − 1.31). The difference in the PP analysis was Δ = 6.9 p.p. (100% vs. 93.1%, respectively), and 1-sided 95% CI equalled (− 2.83, − 1.27). The noninferiority of the 3-day regimen was assumed for Δ not lower than − 8 p.p. and both ITT and PP analyses confirmed the assumed noninferiority of the 3-day PEP.

Since breakthrough influenza was present only in the 7-day group, no further analysis comparing the influenza-related secondary end-points (i.e., need for hospitalization, duration of signs/symptoms, presence of complications) was conducted.

### Safety profile and costs of postexposure prophylaxis

Adverse events possibly related to the drug administration were reported in 21 patients (8 in the 3-day group and 13 in the 7-day group) in the ITT analysis and in 18 patients in the PP analysis (7 and 11 patients, respectively) (Table 2).

The most common adverse event was vomiting reported in 12 cases (3 and 9 cases in the 3-day and 7-day groups, respectively) in the ITT group, and 9 cases (2 and 7 cases, respectively) in the PP group, followed by behavioural changes in 6 (ITT) and 5 patients (PP), and stomachache (n = 4), skin disorders (n = 4 for ITTand n = 3 for PP, and sleep disorders.

Multiple adverse events, i.e. affecting more than one system, were observed in 5 children in the ITT and in 4 cases in the PP analysis.The relative risk of adverse events, or multiple AEs did not differ significantly between the groups (in the ITT analysis or in the PP analysis).

The cumulative costs of the prophylaxis and adverse events management were significantly lower in the 3-day group than in the 7-day group both in the ITT and PP analysis (median 4.5 euro, IQR: 4.5–15.1 versus 10.5 euro, IQR: 10.5–21.1, *p* < 0.01, respectively).

## Discussion

This randomized controlled trial showed that the 3-day regimen of postexposure influenza chemoprophylaxis with oseltamivir is not less efficacious than the 7-day prophylaxis in children hospitalized at the pediatric ward, and decreases the costs of the chemoprophylaxis.

High efficacy of the shorter PEP duration observed in our trial is in line with the results reported by Ishiguro et al. (2016), who showed that a shorter (3-day) PEP may be as effective as the 7 or 10-day regimen^21^. The study included 212 hospitalized patients who had contact with the influenza index cases during their hospitalization; the trial revealed a 93% efficacy when compared to no PEP (95% confidence interval: 53– 99%; *p* = 0.023)^21^. Although the majority of patients were adults, there was a pediatric subgroup (the exact number of children was not specified), which opened the discussion on a possibility of shortening the PEP in children, as well^21^. Similarly, the recent randomized trial from Slovenia aimed to verify the possibility of a shorter PEP regimen by allocating 222 adult patients into 5- or 10-day PEP group; the study confirmed high PEP efficacy with a noninferiority of a shorter PEP course compared to the longer one^26^. There have been no controlled studies which would directly compare differences in hospitalized pediatric population, although a protocol for a prospective, multi-center, single-arm trial has been published and to the best of our knowledge the study is being conducted^27^.

It needs to be emphasized, that PEP efficacy may depend on the study setting and the question of the difference between a household and a nosocomial contact needs to be recognised. Nevertheless, very high efficacy observed in our study is in line with the corresponding studies set in a hospital environment (97% in the study by Shinjoh, 99% in the study by Ishiguro, and 99% in the study by Lepen) and is generally higher than those observed in household settings^21,23,26^. It may be attributed to various factors, including patient’s better compliance, the duration of contact (which may be ceased with a transfer to another room or discharge from the hospital, which is not a routine procedure in the majotiry of household contacts), and the contact quality – contact among patients differs substantially from household contact. In our study, due to the high bed occupancy during the influenza season, transferring the index cases immediately after the diagnosis of influenza was not possible, similarly, transferring the patients who have had contact and are possibly shedding the virus is not a routine, rather, the method of choice is the cohortation of patients. All efforts to limit the duration of contact are being made at our ward, and patients newly diagnosed with influenza are transferred to other patient rooms, depending on the room availability. Nevertheless, no analysis on the correlation with the contact duration has been made, since the incubation period varies hugely among the patients and the first day of the reported syndromes is not the first day of the infectiousness; also shedding in children is more heterogeneous than in adults, and it may also last longer due to a slower decline rate^28^. A study by Lau (2010) proved influenza A shedding a day before the acute respiratory infection onset and 1–2 days in the case of influenza B, thus, in accordance with Ishiguro et al. , we had assumed that calculating true duration of exposition to the influenza virus may be seriously misleading^21,29^

In our series of patients, two failures in the prophylaxis took place in the 7-day regimen group, with no failures in the 3-day group; however, it may be attributed only to the randomization, not the duration of the PEP itself. One patient had contact and was infected with influenza type A, and the other one with type B, while the PEP failures observed in the study by Shinjoh et al. , the only fully pediatric study, were seen in two patients who had contact with influenza type B^23^. It should thus be remembered that both influenza A and B need to be considered a significant threat^30,31^.

A crucial factor for PEP efficacy seems to be appropriate timing. Its significance is reflected universally in the recommendations on the PEP, which is not advised if more than 48 h have passed since the first exposition to the index case^32^. The PEP failures in the study by Shinjoh et al. developed in patients who were given oseltamivir more than 24 h after the contact, while in our study each patient was given oseltamivir within 12 h after the first contact had been recognized, thus, the PEP failures cannot be attributed to a delay in the PEP^23^.

The patients’ vaccination status did not influence the effects of the PEP in our group either; none of the contacts had ever been vaccinated against influenza, neither were the mothers of infants vaccinated during the pregnancy. A problem of the low influenza vaccine uptake (approximately 2.6% in the general population, and 0.85% in children under 14 years of age) is a huge public health concern in Poland, alike in other countries^33,34^.,

The most frequent oseltamivir adverse event (AE) observed in this study was nausea/vomiting; metaanalyses on the NAIs use had previously reported vomiting as the only statistically significant AE related to the use of oseltamivir^20,35^. Interestingly, the frequency of nasusea and vomiting increased with the number of doses in adult patients^36^. In our patients, more cases of vomiting were observed in the 7-day group, although the relative risk was insignificant, alike other adverse events relative risks. Although the lack of statistical significance needs to be verified in a broader group of patients (the group size is relatively small), the tendency towards a higher frequency of AEs in the 7-day PEP group observed in our study may possibly be explained by the duration of the PEP, which increases the total number of doses. The withdrawal due to AEs was scarce and reached 5.1%; it took place in patients assigned to both the 3- and 7-day groups (on 2nd, 3rd and 4th day of prophylaxis), and no cases of influenza were reported. In the previous studies on oseltamivir treatment (where the dose was twofold higher than in the case of PEP), the withdrawal from treatment, as well as withdrawal due to AEs, was statistically insignificant as compared to placebo^20^.

Decreasing the exposition of patients to the drug is crucial in order to reduce the risk of adverse events, and, even more importantly, to minimize the risk of a drug resistance development. Although, according to the CDC data, a resistance to NAIs is relatively uncommon, it remains a possible threat to the public health, and drug resistance cases have been described in young children^32,37^. The patients who developed influenza during oseltamivir prophylaxis in our study were then successfully treated with oseltamivir, and the dose effect might be a possible explanation, since prophylaxis means administering half of the actual treatment dose.

There are also certain limitations to this study. First, the number of patients enrolled was relatively small, nevertheless, this is a pilot study and brings important data, especially considering the scarce pediatric studies in this area. The recommended minimal sample size for a two-arm pilot trial varies between 24 and 70, depending on the sources , and the majority of authors suggests the number of up to 55^38–40^. Second, this is only a single-centre study, and broader multi-centre analyses are required. Third, the patients (and caregivers) were not blinded and an open model of the study might result in a possible bias, but patients underwent everyday examination during the hospitalization, and after a hospital discharge in the follow-up period caregivers were directly asked about any symptoms in their children. Also, we did not analyse the various exposition patterns, including the patients’ hygienic or social behaviour during the hospitalization, yet, due to other differences (e.g., the varying infectiousness of the influenza strains, the correlation with the number of virus copies). We did not analysed visitors as potential source of influenza, although due to epidemiological concerns during high respiratory infections seasons, the number of visitors is limited to one single person taking care of a child. No in-depth analysis on influenza type or subtype/lineage was performed due to the small number of patients, yet only extremely large trials could answer this question.

In conclusion, this study shows noninferiority of 3-day postexposure prophylaxis with oseltamivir compared to 7-day PEP with high efficacy of both regimens investigated. Certainly, as a pilot study, this thesis needs further verification in large numbers of patients enrolled in multi-site trials. Further studies will need to confirm the efficacy of a shorter PEP regimen, establish its benefits, including cost-effectiveness, and probably verify an optimal duration of PEP. However, based on the promising efficacy and safety profile, a shorter course of postexposure prophylaxis may help to achieve better drug tolerability and reduce the risk of developing drug resistance.

## Data Availability

The datasets generated during and/or analysed during the current study are not publicly available due to legal reason, but are available from the corresponding author on reasonable request.
